# Rapid Tissue‐CSF Water Exchange in the Human Brain Revealed by Magnetization Transfer Indirect Spin Labeling

**DOI:** 10.1002/mrm.70298

**Published:** 2026-02-09

**Authors:** Yihan Wu, Kexin Wang, Licheng Ju, Anna Li, Qin Qin, Feng Xu, Doris D. M. Lin, Lawrence Kleinberg, Jiadi Xu

**Affiliations:** ^1^ Department of Biomedical Engineering Johns Hopkins University Baltimore Maryland USA; ^2^ F.M. Kirby Research Center for Functional Brain Imaging Kennedy Krieger Research Institute Baltimore Maryland USA; ^3^ Russell H. Morgan Department of Radiology and Radiological Science Johns Hopkins University School of Medicine Baltimore Maryland USA; ^4^ Department of Radiology University of Washington Seattle Washington USA; ^5^ Department of Radiation Oncology and Radiation Molecular Sciences Johns Hopkins University Baltimore Maryland USA

**Keywords:** cerebrospinal fluid (CSF), edema, glymphatic system, magnetization transfer (MT), magnetization transfer indirect spin labeling (MISL), perivascular spaces (PVS), tissue‐CSF water exchange, tumor

## Abstract

**Purpose:**

To apply the Magnetization Transfer Indirect Spin Labeling (MISL) MRI technique for quantifying tissue‐CSF water exchange in the human brain, and to investigate its utility in (1) evaluating tissue‐CSF water exchange within perivascular spaces (PVS), and (2) characterizing altered water exchange dynamics in pathologic conditions.

**Methods:**

MISL was implemented on a 3 T MRI using off‐resonance magnetization transfer to label parenchymal water. The resulting exchange with CSF was captured via long‐TE 3D‐TSE readout to suppress parenchymal signals. CSF‐region‐specific quantification was achieved by atlas‐based segmentation. Studies were conducted in healthy subjects across age groups and in patient with metastatic brain tumor.

**Results:**

MISL revealed widespread and regionally heterogeneous tissue‐CSF exchange, with the strongest signals observed in the PVS and areas adjacent to the choroid plexus. MISL signals were typically 2%–3% in the ventricles and subarachnoid space, and reached 3% in the cerebellar regions, suggesting tissue‐to‐CSF flow (TCF) in the range of 100–300 mL/100 mL/min. The high MISL signals observed in the PVS (∼8.4%) indicated active tissue‐CSF water exchange, providing functional information of the PVS that conventional T2w imaging cannot capture.

Significant age‐dependent declines in TCF were observed across most brain regions, except for the third and fourth ventricles. In the tumor patient, MISL revealed elevated water exchange, even where no overt FLAIR hyperintensity was present.

**Conclusion:**

MISL enables robust, non‐invasive mapping of tissue‐CSF exchange with high sensitivity and spatial resolution. MISL provides a unique window into tissue‐CSF exchange within PVS, which may reflect glymphatic function.

## 1 Introduction

Cerebrospinal fluid (CSF) is essential to the central nervous system, playing a critical role in cushioning the brain against mechanical impact, regulating chemical homeostasis for optimal neuronal function, facilitating the removal of metabolic waste, and distributing essential nutrients [1]. For over a century, the conventional understanding of CSF production centered on the choroid plexus (CP), composed of a network of capillaries and ependymal cells [2, 3, 4, 5, 6, 7, 8]. It was widely believed that CSF was primarily produced through the filtration of blood plasma at the CP. However, despite decades of research, the precise mechanisms of CSF production, circulation, and clearance remain a subject of ongoing debate, particularly with the emergence of new imaging techniques and conceptual models [9, 10, 11]. These findings indicate that CSF production and movement involve multiple pathways beyond the CP, with additional contributions from ependymal cells lining the ventricular walls. Moreover, the discovery of the glymphatic system has further increased the complexity of our understanding of CSF circulation [12, 13, 14, 15]. The glymphatic system suggests a more dynamic exchange between CSF and interstitial fluid (ISF). This interaction highlights the possibility that CSF circulation is not merely a passive bulk flow process but rather an active and regulated exchange influenced by physiological and pathological conditions. The uncertainty surrounding the exact mechanisms of CSF circulation has significant implications for understanding and managing CSF‐related disorders, such as normal pressure hydrocephalus, idiopathic intracranial hypertension, and Alzheimer's disease.

Stimulated by the discovery of the glymphatic system, a variety of non‐invasive MRI techniques have been extensively developed to image CSF dynamics in the brain [16]. Among these, phase‐contrast MRI [17, 18, 19], diffusion‐weighted MRI [20, 21, 22], intravoxel incoherent motion [23, 24], spin‐labeling methods [25, 26, 27], and fMRI methods [28] have been widely used to visualize bulk CSF flow, particularly within the ventricles. However, these techniques primarily focus on bulk CSF movement and provide limited insight into the water exchange processes occurring between CSF and surrounding brain tissues. Tissue‐CSF exchange is still mainly investigated by MRI contrast agents, such as intracranial [11, 13, 29, 30, 31] or intrathecal injection [32, 33, 34] of gadolinium‐based contrast agents, as well as intravenous D‐glucose infusion [35, 36]. While effective, these contrast‐based MRI approaches are far from ideal for routine and repeated measurements in clinical settings due to safety concerns and logistical challenges. A major breakthrough in non‐invasive CSF imaging came with the development of arterial spin labeling (ASL) MRI, the first non‐invasive method capable of detecting CSF production from blood [37]. ASL techniques have demonstrated that CSF is produced not only in the CP but also in the subarachnoid space (SAS) surrounding the cortex in the human brain [38]. Other spin‐labeling MRI methods have emerged to further characterize tissue‐CSF exchange, including Magnetization Transfer Indirect Spin Labeling (MISL) [39, 40] and Phase Alternate Labeling with Null Recovery (PALAN) [41]. These methods leverage the significant differences in semi‐solid macromolecule concentration, diffusion, and T_1_/T_2_ relaxation times between CSF and brain parenchyma to track water exchange across compartments. Using these advanced techniques, rapid tissue‐CSF water exchange in the mouse brain ventricles was discovered, findings that were later confirmed in human studies using a novel T_2_‐labeling approach [42] and MISL with on‐resonance magnetization transfer (MT) labeling approach, that is, on‐resonance MISL [43].

These advancements mark a significant shift in our understanding of CSF circulation and its interaction with brain tissues, opening new possibilities for investigating neurological diseases associated with CSF dysfunction. Further refinement of these non‐invasive MRI techniques will be essential for translating these discoveries into clinical applications. Among these techniques, MISL has demonstrated significantly higher sensitivity (> 10% water signal in mouse brain) [39, 40] compared to the T_1_/T_2_‐based approaches, making it a promising method for detecting tissue‐CSF water exchange non‐invasively. However, despite its promise, MISL based on off‐resonance MT labeling has not yet been developed or validated for human brain imaging. In particular, the perivascular space (PVS), which is not clearly visible in rodent models, has not been explored using either off‐resonance or on‐resonance MISL techniques. In this study, we aimed to optimize the off‐resonance MISL technique for human brain applications and establish a quantitative framework for measuring tissue‐CSF water exchange in the SAS, ventricles, and PVS. To investigate the utility of MISL in characterizing early physiological changes and altered tissue‐CSF dynamics under pathological conditions, we examined age‐related variations in tissue‐CSF water exchange and evaluated MISL in assessing water exchange processes in brain tumors and tumor‐associated edema.

## 2 Methods

### 2.1 Participants

A total of 12 healthy volunteers (age: 41 ± 20 years; 5 females, 7 males) and one patient with a left peri‐Sylvian arachnoid cyst participated, and the specific number of participants for each study is detailed below. Scanning procedures were conducted on a Philips MR Ingenia Elition 3.0 T scanner (Philips Healthcare, Best, The Netherlands), utilizing a quadrature body transmit coil and a 32‐channel receive head coil. One 64‐year‐old female with brain metastases was scanned on Philips 3.0 T Achieva, with the same parameters. Ethical approval was obtained from the Johns Hopkins Medicine Institutional Review Board (IRB), and all participants have provided their informed consent.

### 2.2 MISL Sequence

**FIGURE 1:**
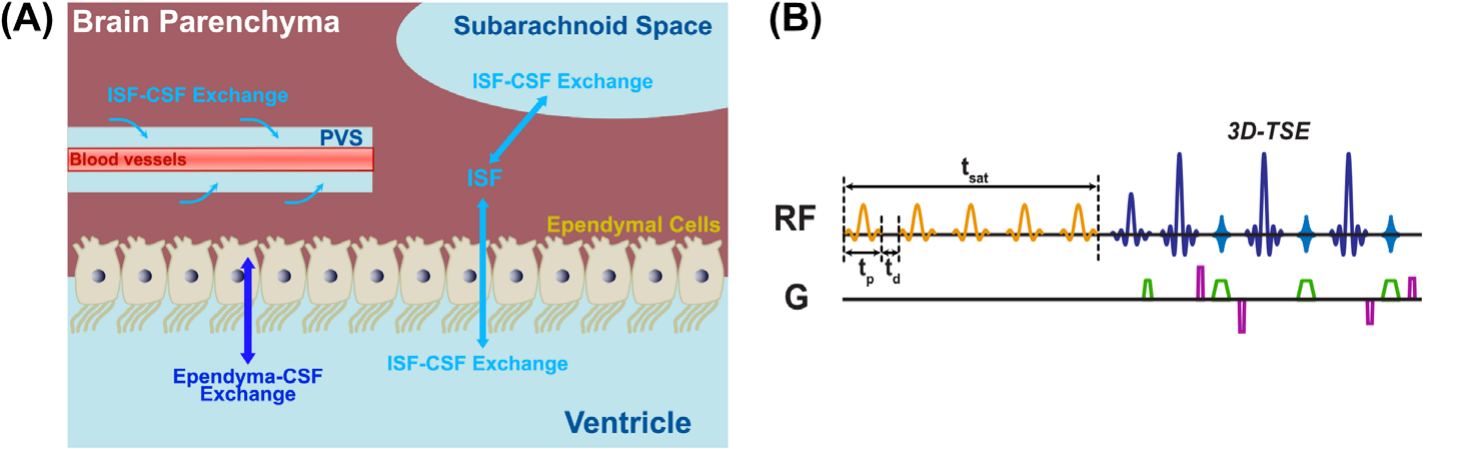
(A) Schematic illustration of the potential sources contributing to the MISL signal across the brain, including interstitial fluid (ISF)‐CSF exchange in the perivascular space (PVS) and subarachnoid space, and ependyma‐CSF and ISF‐CSF exchange in the ventricles. (B) Diagram of the MISL pulse sequence. Frequency‐selective magnetization transfer labeling pulses are applied to saturate water in the parenchyma, followed by a long‐TE 3D turbo spin‐echo (3D‐TSE) readout to selectively image CSF while attenuating parenchymal and blood contributions.

As illustrated in Figure 1A, the MISL sequence selectively labels the water in brain parenchyma via MT, while leaving CSF unaffected due to its negligible semi‐solid macromolecule content. Labeled water molecules in the brain parenchyma subsequently exchange with CSF, which is captured as a reduction in CSF signal intensity.

The MISL saturation module (Figure 1B) consists of a train of frequency‐selective MT pulses, which is optimized to maximize labeling efficiency for parenchyma, while minimizing CSF water direct saturation (DS). In the current study, a train of SincGaussian pulses (number of pulses: N_pulse_ = 50) with a peak RF amplitude of 3 μT (equivalent to 1.90 μT continuous wave), a pulse width (t_p_) of 50 ms, and an interpulse delay (t_d_) of 25 ms was used, that is, total saturation time (t_sat_) 3.725 s. A frequency offset of −10 ppm was used for labeling unless specified, and 200 ppm for control images.

A 3D Turbo Spin‐Echo (3D‐TSE) readout was used for image acquisition. The MRI signal from parenchyma was attenuated by a long‐TE to exploit the difference in T2 relaxation times (parenchyma < 100 ms vs. CSF > 1000 ms). The imaging parameters were as follows: field of view (FOV) = 160 × 183 × 150 mm^3^; slice number = 75; acquisition matrix = 80 × 91 × 75; reconstruction matrix size = 256 × 256 × 75; acquisition resolution = 2 × 2 × 2 mm^3^; TSE factor = 240 with linear ordering and startup echoes of 7; refocusing angle = $120^{{}^{\circ}}$; compressed sensing (CS) factor = 8; echo spacing = 8.5 ms; TR/effective TE = 8 s / 1081 ms. Control and label acquisitions were repeated 18 times for averaging, yielding a total scan duration of 14.5 min (An alternative TSE readout with a startup echoes of 30 (240 ms) was also evaluated to assess potential contamination of residual tissue signal within the PVS).

Labeling efficiency in the parenchyma, that is, the parenchyma MT, was measured for each subject with identical MT saturation pulses and a single slice 2D‐TSE readout (FOV = 160 × 160 × 5 mm^3^; resolution = 2 × 2 × 5 mm^3^; TSE factor = 40 with low‐high ordering; refocusing angle = 120°; SENSE factor (P direction) = 2; TR/TE = 6 s / 35 ms). Two pairs of control/label images were acquired, with a scan duration of 0.5 min. Labeling efficiency in all tissues was assumed equivalent to the measured parenchymal MT. To quantify the water relaxation rate of parenchyma in the rotating frame (*R*_1ρ,tissue_), the MISL sequence was repeated using the same 2D‐TSE readout parameters as in the parenchyma MT studies, while varying N_pulse_ (N_pulse_ = 7, 14, 27, 40, 50). As a consequence, t_sat_ = 500, 1025, 2000, 2975, and 3725 ms were achieved. The mean MT signal ($∆Z^{\text{Tissue}}$) within the whole brain parenchyma in the selected slice was defined by

(1) $$∆Z^{\text{Tissue}}=\left(S_{\text{Control}}^{\text{Tissue}}-S_{\text{Label}}^{\text{Tissue}}\right)/S_{\text{Control}}^{\text{Tissue}}$$

where $S_{\text{Control}}^{\text{Tissue}}$ and $S_{\text{Label}}^{\text{Tissue}}$ are tissue signals from the 200 ppm and −10 ppm offset acquisitions, respectively. The dependence of $∆Z^{\text{Tissue}}$ on t_sat_ was modeled using a mono‐exponential recovery function to determine R_1ρ,tissue_ [44]:

(2) $$∆Z^{\text{Tissue}}\left(t\right)=\alpha \cdot \frac{\mathrm{TCF}}{6000}\cdot \left(1-e^{-R_{1\rho ,\text{tissue}}\cdot t_{\mathrm{sat}}}\right)$$

where $\alpha =\Delta Z_{\mathrm{SS}}^{\text{Tissue}}$ is the steady‐state tissue MT signal, that is, labeling efficiency. The tissue‐to‐CSF flow (TCF) rate is expressed in units of tissue water volume delivered per 100 unit volume of CSF per minute, mL/100 mL/min.

### 2.3 Parameter Optimization

To achieve maximum labeling efficiency, full Z‐spectra of the brain parenchyma were acquired by applying the same MT saturation module and the same single slice 2D‐TSE readout across a range of frequency offsets (−20 to 20 ppm, step size = 1 ppm). A total of 3 subjects were included for this optimization. Four M_0_ reference images at 200 ppm were acquired and averaged for normalization. The peak saturation power (B_1_) for the saturation pulses was varied from 1 to 4 μT in 1 μT increments. The extent of CSF DS resulting from the MT preparation was simulated using Bloch equation modeling with only the CSF pool, assuming a T1 of 4350 ms and a T2 of 2000 ms for CSF [45]. In addition, MISL sequences were acquired on three subjects using frequency offsets of ±10, ±5, ±4, ±3, −15, and −20 ppm at 3 μT peak RF amplitude, each with six pairs of control/label images to further characterize the CSF DS.

### 2.4 Image Processing and Analysis

Similar to the tissue MT measurement in Equation (1), the MISL signal was calculated by subtracting the label $S_{\text{Label}}$ (offset −10 ppm) from control $S_{\text{Control}}$ (offset 200 ppm) images, yielding voxelwise maps of ΔZ given by

(3) $$∆Z=\left(S_{\text{Control}}-S_{\text{Label}}\right)/S_{\text{Control}}$$

The TCF can then be determined by: [39].

(4) $$\mathrm{\Delta Z}=\alpha \cdot \frac{\mathrm{TCF}}{6000}\cdot \frac{1}{R_{1,\mathrm{CSF}}}\left(1+e^{-R_{1\rho ,\text{tissue}}\cdot t_{\mathrm{sat}}}\cdot \frac{R_{1,\mathrm{CSF}}}{R_{1,\mathrm{app}}}-e^{-R_{1,\mathrm{CSF}}\cdot t_{\mathrm{sat}}}\cdot \frac{R_{1\rho ,\text{tissue}}}{R_{1,\mathrm{app}}}\right)$$

where $R_{1,\mathrm{app}}=R_{1\rho ,\text{tissue}}-R_{1,\mathrm{CSF}}$ and $R_{1\rho ,\text{tissue}}$ was measured experimentally using Equation (2). A detailed description of the MISL quantification model is provided in the Supporting Information. Three types of images can be generated from the MISL experiment: ΔS images—the difference between control and label images, calculated as $\mathrm{\Delta S}=S_{\text{control}}-S_{\text{label}}$; ΔZ images—the normalized difference, calculated as $\mathrm{\Delta Z}=\mathrm{\Delta S}/S_{\text{control}}$ and TCF maps calculated from ΔZ images using Equation (4). In the calculation, the measured $R_{1\rho ,\text{tissue}}$ and a literature‐based value for $R_{1,\mathrm{CSF}}=0.23s^{-1}$ were used [45]. Both $R_{1\rho ,\text{tissue}}$ and $R_{1,\mathrm{CSF}}$ were assumed to be identical across all subjects, while *α* was individually determined for each subject as $\alpha \approx ∆Z^{\text{Tissue}}\left(3.725s\right)$.

To define regional measures of the MISL signal, T1‐weighted (T1w) anatomical images from MPRAGE were acquired for image segmentation and reference space definition. The MPRAGE sequence parameters were as follows: FOV = 160 × 183 × 150 mm^3^; resolution = 1 × 1 × 1 mm^3^; TR/TE = 8.0/3.7 ms; TFE factor = 96 with linear ordering; flip angle = 8°, SENSE factor (P direction) = 2. The total scan duration of MPRAGE was 2.5 min. High‐resolution long‐TE T2w images were also acquired with TSE to generate PVS masks, with the following parameters: FOV = 160 × 183 × 150 mm^3^; resolution = 1 × 1 × 1 mm^3^; TSE factor = 240 with linear ordering and startup echoes of 57; refocusing angle = $120^{{}^{\circ}}$; CS factor = 8; echo spacing = 8.5 ms; TR/TE = 2946/1138 ms.

**FIGURE 2:**
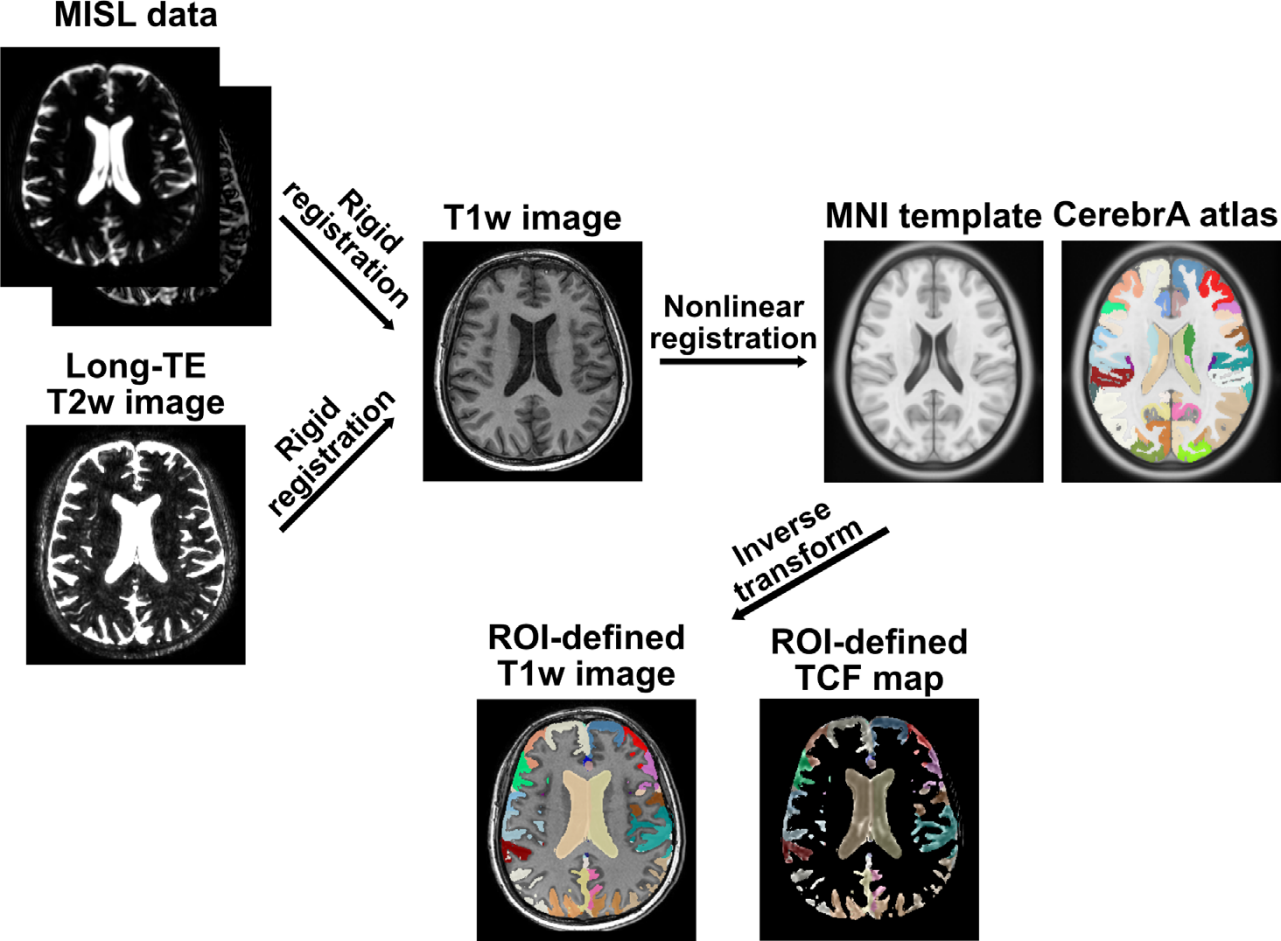
Image processing pipeline for regional tissue‐CSF exchange analysis.

The CSF segmentation pipeline follows a similar procedure as previously described [42] and is illustrated in Figure 2. Each subject's T1w image was nonlinearly registered to the MNI‐2009 symmetric template using the ANTs SyN framework, and deformation fields and affine transforms were inverted to propagate CerebrA atlas labels into subject space. FSL's MCFLIRT tool was used for motion correction of the MISL images. To register MISL data, the average of all control and label images were rigidly aligned to the T1w images, and the same transformation was applied to the MISL ΔZ maps to bring them into the T1w anatomical space. TCF maps were subsequently calculated from the ΔZ maps. Similarly, long‐TE T2w images were also rigidly registered to the same T1w space to ensure alignment for PVS masking. A CSF mask was generated by thresholding the average control image at an empirically determined intensity cutoff to robustly isolate CSF compartments. To delineate regions expected to exhibit tissue‐CSF exchange, atlas ROIs were morphologically dilated and intersected with the CSF mask. The defined ROIs are further grouped into composite categories representing the lateral ventricle, third ventricle, fourth ventricle, cerebral SAS, cerebellar SAS, and CSF around the limbic system. CP was segmented manually by the same person.

For the PVS mask, brain extraction was first performed on T1w images using FSL's BET tool. The brain mask was applied to high‐resolution long‐TE images to exclude the extracranial signal. High‐intensity voxels were then thresholded, and connected component analysis was used to remove large CSF structures. The remaining voxels were classified as PVS and applied to the ΔZ and TCF maps to extract corresponding values.

Quantitative analyses were performed to assess spatial variability and age‐related trends in MISL TCF. For each subject, mean ΔZ and TCF values were calculated for each anatomically defined ROI and composite region. To assess age‐related effects, linear regression models were fitted to mean TCF values within composite compartments, including the lateral ventricle, third ventricle, fourth ventricles, cerebral SAS, cerebellar SAS, CSF around the limbic system, and the whole‐brain CSF region. Statistical analyses were conducted using R software.

To assess the reproducibility of the MISL measurement, we re‐scanned three volunteers (age: 38 ± 24 years; 2 females, 1 male). The MISL sequence was repeated under identical acquisition parameters, and all data were processed using the same analysis pipeline. For each subject, the ΔZ and TCF values were extracted from each atlas‐based ROI, and test–retest comparisons were performed across all ROIs.

To assess the utility of MISL in evaluating water exchange processes in brain tumors and tumor‐associated edema. We applied MISL to a patient with brain metastases to assess water exchange and accumulation in tumors and surrounding tissue, where there is disruption of the blood–brain barrier and vasogenic edema.

## 3 Results

### 3.1 Parameter Optimization

**FIGURE 3:**
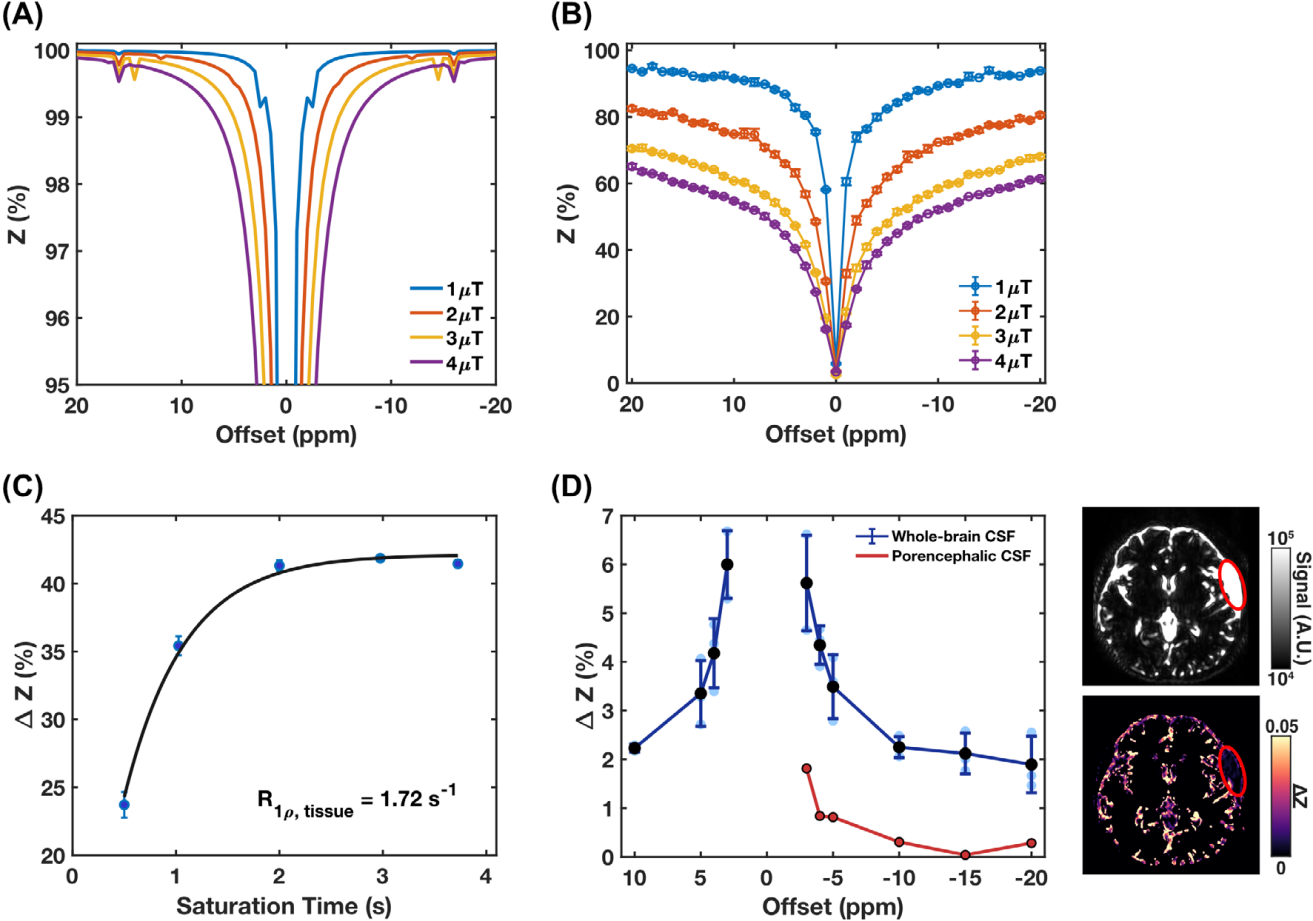
Optimization of MISL labeling parameters. (A) Simulated Z‐spectrum of CSF with only water pool. (B) Experimental Z‐spectra of brain parenchyma for the selected slice averaged across subjects (*n* = 3). Saturation offsets were swept from −20 to +20 ppm in 1 ppm increments. (C) Determination of the water relaxation rate in the rotation frame (R_1ρ,tissue_). The plot shows the mean ΔZ signal within brain parenchyma (*n* = 2) for the selected slice as a function of saturation time (500, 1025, 2000, 2975, and 3725 ms). (D) Averaged MISL ΔZ signals (*n* = 4) from the whole‐brain CSF (blue) and peri‐Sylvian arachnoid cyst (red) as a function of offset. The control image and ΔZ map from the subject with a left peri‐Sylvian arachnoid cyst are shown, with the peri‐Sylvian arachnoid cyst indicated by a red ellipse.

Simulations of the CSF Z‐spectrum (Figure 3A) demonstrated that DS was negligible at a frequency offset of ±10 ppm (< 0.26%) using the MT labeling scheme applied in this study, with a peak B_1_ of 3 μT. Experimental Z‐spectra from brain parenchyma (Figure 3B) confirmed that MT effects increased with rising B_1_ amplitude, showing a sharp increase from 1 to 3 μT (MT = 10.69% to 41.59%), and only a modest additional gain at 4 μT (47.90%). Therefore, a peak B_1_ of 3 μT was selected for use in subsequent studies unless otherwise specified.

The $R_{1\rho ,\text{tissue}}$ measurement results are shown in Figure 3C, where the tissue MT signal ($∆Z^{\text{Tissue}}$) increased with a longer t_sat_ and reached a plateau around 2–4 s. A $R_{1\rho ,\text{tissue}}$ value of 1.72 s^−1^ was obtained through least‐squares fitting using Equation (2), with *α* = 0.42. Notably, the $∆Z^{\text{Tissue}}$ value at t_sat_ = 3.725 s ($∆Z^{\text{Tissue}}\left(3.725s\right)$= 0.42) was already close to *α*. Therefore, $\alpha =∆Z^{\text{Tissue}}\left(3.725s\right)$ will be used to calculate the TCF in subsequent analyses.

Whole‐brain CSF ∆Z values were also evaluated across a range of frequency offsets (Figure 3D). Within the central offsets between −5 and +5 ppm, ∆Z increased rapidly, which suggests substantial contamination from DS. In contrast, offsets beyond ±10 ppm showed a much weaker offset dependence, consistent with the expected $∆Z^{\text{Tissue}}$ pattern in Figure 2B and indicating minimal DS contribution—an observation in agreement with the simulation results in Figure 2A. To experimentally quantify the DS contamination, MISL was performed on a subject with a left peri‐Sylvian arachnoid cyst, a cavity filled exclusively with CSF and lacking tissue‐CSF exchange. The DS signal measured in the peri‐Sylvian arachnoid cyst was only 0.30% (Figure 3), confirming that DS is negligible compared to the MISL signal (> 2%) under the current MT labeling protocol. This value is also close to the simulation result (0.26%).

With a peak B_1_ of 3 μT, using a lower offset frequency (< 5 ppm) yields high labeling efficiency but also introduces significant DS in CSF (Figure 3). Consequently, a strong MISL signal will appear in CSF—even in the absence of water exchange or measurable protein content—which can contaminate the measured MISL effect. Based on these results, a saturation offset of −10 ppm was selected as an optimal balance between labeling efficiency and DS suppression, making the protocol suitable for mapping tissue‐CSF water exchange in regions with both fast and slow dynamics.

### 3.2 Whole‐Brain Tissue‐CSF Water Exchange Mapping

**FIGURE 4:**
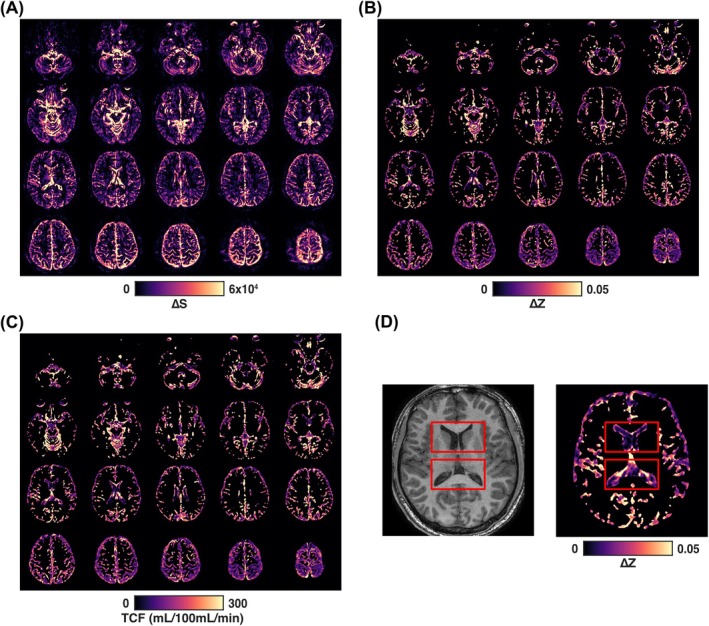
(A) Representative MISL ΔS map from a healthy volunteer, illustrating regional tissue‐CSF water exchange across the brain. (B) Corresponding ΔZ maps from the same subject by applying a CSF mask, reflecting the normalized exchange‐sensitive signal. (C) Corresponding TCF maps derived from the ΔZ maps for the same subject using Equation (4). (D) Zoomed‐in T1w images and corresponding ΔZ map showing minimal signal in the center of the lateral ventricle and elevated signal at the ventricle‐tissue interface, particularly around the choroid plexus.

Representative MISL ΔS, ΔZ, and TCF maps from a typical healthy volunteer are shown in Figure 4. The ΔS maps (Figure 4A) revealed spatially heterogeneous exchange signal predominantly along the ventricular borders, cereberal SAS, cerebellar SAS, and CP, consistent with tissue‐CSF water exchange occurring at these interfaces. The corresponding ΔZ maps (Figure 4B) and TCF maps (Figure 4C) further highlighted CSF regions adjacent to tissue—including periventricular, cereberal and cerebellar SAS, and the CP—demonstrating clear delineation with elevated exchange signal. Notably, in the zoomed‐in images (Figure 4D), a minimal ΔZ signal was observed in the central region of the lateral ventricle, indicating effective suppression of CSF DS with current labeling. In contrast, strong ΔZ signals were clearly visible around the CP.

### 3.3 Assessment of Tissue‐CSF Water Exchange in PVS With MISL

**FIGURE 5:**
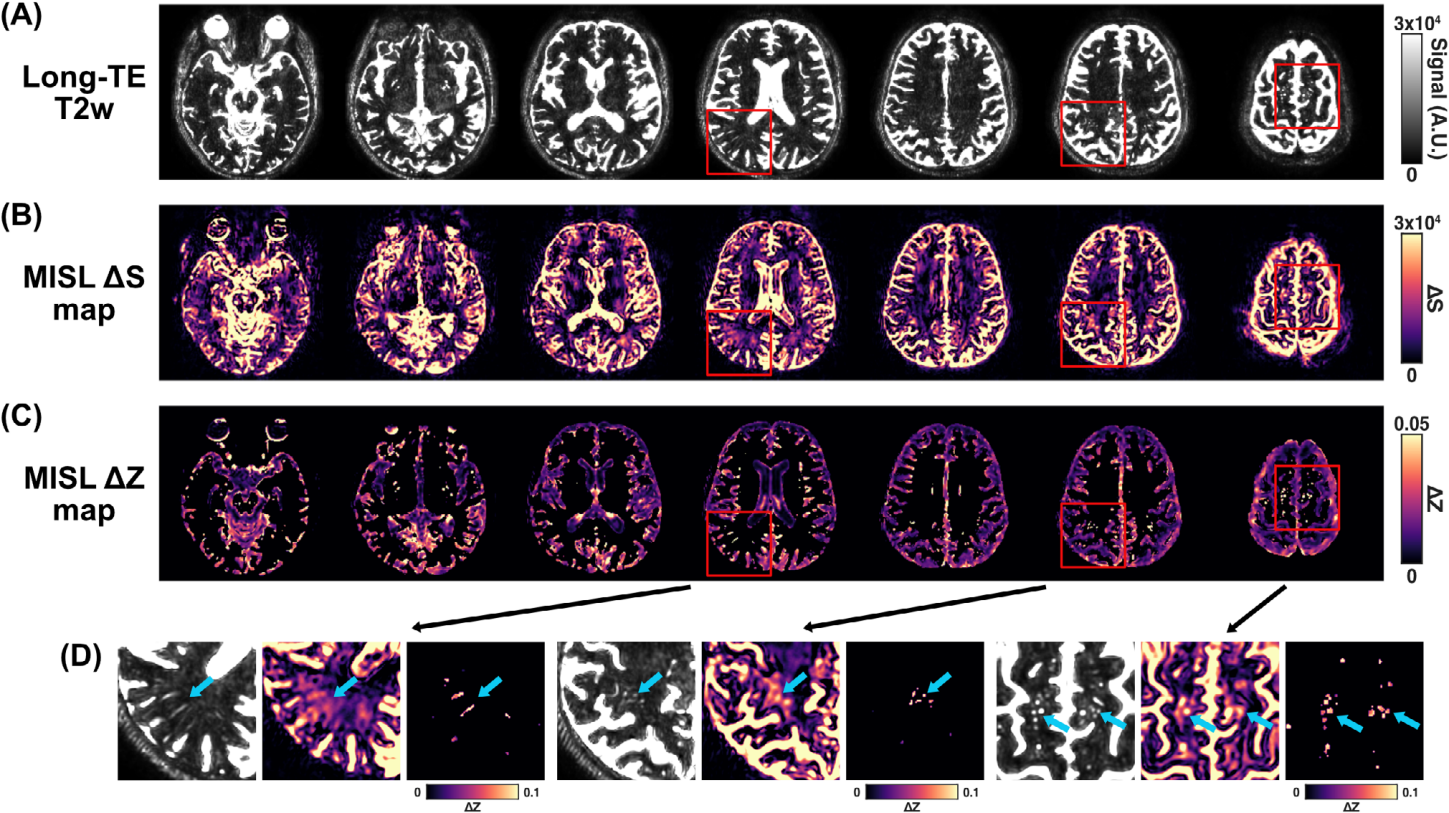
High‐resolution long‐TE T2w, MISL ΔS, and MISL ΔZ in a typical healthy subject. (A) Long‐TE T2w images delineate anatomical PVS in the basal ganglia and the centrum semiovale. (B) MISL ΔS maps show markedly enhanced MISL signal along the same PVS trajectories. (C) MISL ΔZ maps quantify tissue‐CSF flow rate. (D) Zoomed views highlight PVS: Long‐TE T2w shows PVS anatomy, ΔS shows contrast sensitive to tissue‐CSF water exchange, and ΔZ within the PVS mask quantifies tissue‐CSF flow rate. PVSs are indicated by teal arrows.

Figure 5 shows long‐TE T2w images and corresponding MISL ΔS and ΔZ maps acquired from a healthy subject. The long‐TE T2w images (Figure 5A) depict detailed anatomical CSF structures, including the ventricles, SAS, and PVS. The MISL ΔS maps (Figure 5B) provide complementary contrast sensitive to tissue‐CSF water exchange. The MISL ΔZ maps (Figure 5C) reflect the normalized exchange‐sensitive signal. The zoomed‐in views (Figure 5D) highlight the locations of prominent PVSs. The most striking group of PVSs is seen in the basal ganglia, corresponding to spaces surrounding the lenticulostriate arteries. The second most noticeable PVS group appears in the centrum semiovale, following the course of medullary perforating arteries. Corresponding MISL ΔS maps show markedly enhanced signal along the same PVS trajectories, reflecting active water exchange. The MISL ΔZ maps with the PVS mask quantify tissue‐CSF water exchange within PVS. This suggests that while conventional T2w imaging can capture PVS structure, MISL can reveal functional information of PVS not visible through static fluid contrast alone.

### 3.4 Regional Variations in Tissue‐CSF Water Exchange

**FIGURE 6:**
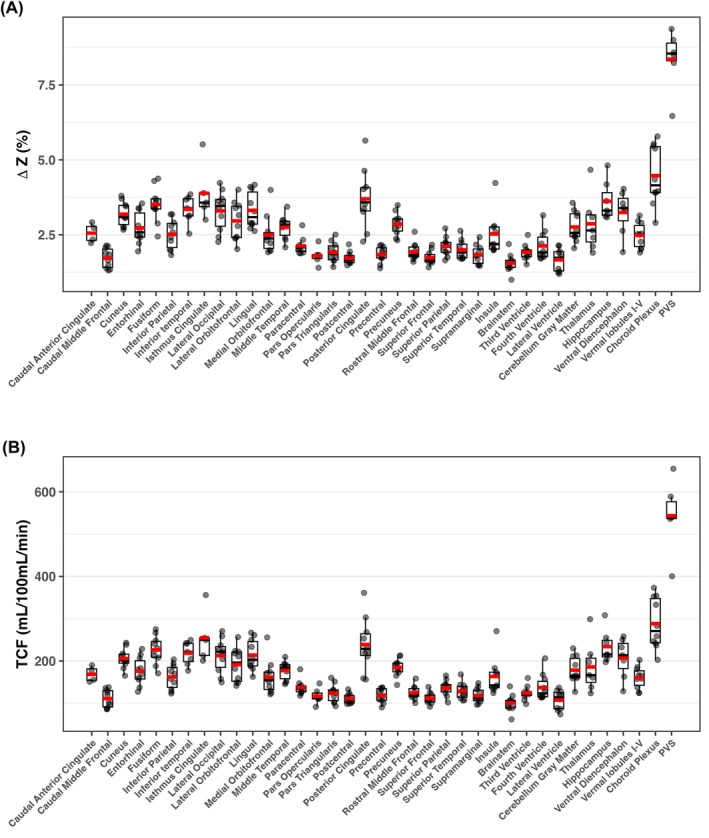
Boxplots of MISL ΔZ (A) and exchange rate (TCF) (B) across subjects (*n* = 10), evaluated using the CerebrA atlas, as well as perivascular space (PVS) and manually segmented regions of the choroid plexus. The strongest MISL signals are observed in the choroid plexus and PVS.

Analysis of MISL‐derived signals revealed marked spatial heterogeneity in tissue‐CSF water exchange across ventricles, SAS and PVS (Figure 6). The PVS demonstrated the highest TCF (ΔZ = 8.36%, TCF = 543 mL/100 mL/min), followed by the CP (ΔZ = 4.47%, TCF = 288 mL/100 mL/min), both of which are key components of fluid exchange pathways in the brain.

High ΔZ and TCF values were observed in CSF regions adjacent to limbic and posterior cortical areas, including the isthmus cingulate (ΔZ = 3.89%, TCF = 255 mL/100 mL/min), posterior cingulate (3.70%, 239 mL/100 mL/min), and hippocampus (3.62%, 234 mL/100 mL/min). Similar high exchange was found in CSF near visual and temporal cortices, such as the fusiform, lingual, lateral occipital, and inferior temporal regions, all showing ΔZ > 3.30% and TCF > 213 mL/100 mL/min. These patterns suggest enhanced exchange at interfaces between brain parenchyma and CSF, particularly in posterior and medial brain regions.

Subcortical‐adjacent CSF regions, including those near the ventral diencephalon (ΔZ = 3.25%, TCF = 207 mL/100 mL/min) and thalamus (ΔZ = 2.87%, TCF = 186 mL/100 mL/min), also exhibited relatively high exchange. Adjacent CSF spaces near the precuneus, lateral orbitofrontal, entorhinal cortex, and cerebellar gray matter showed moderately elevated ΔZ (2.7%–3.0%) and TCF (∼176–191 mL/100 mL/min).

In contrast, CSF regions adjacent to primary sensorimotor and frontal cortices exhibited the lowest exchange signals. These included areas near the precentral, postcentral, supramarginal, and superior frontal gyri, where ΔZ was typically < 2% and TCF < 129 mL/100 mL/min. Ventricular CSF compartments such as the lateral, third, and fourth ventricles, as well as the brainstem‐adjacent CSF, also demonstrated low ΔZ and TCF values.

### 3.5 Test–Retest Reproducibility

**FIGURE 7:**
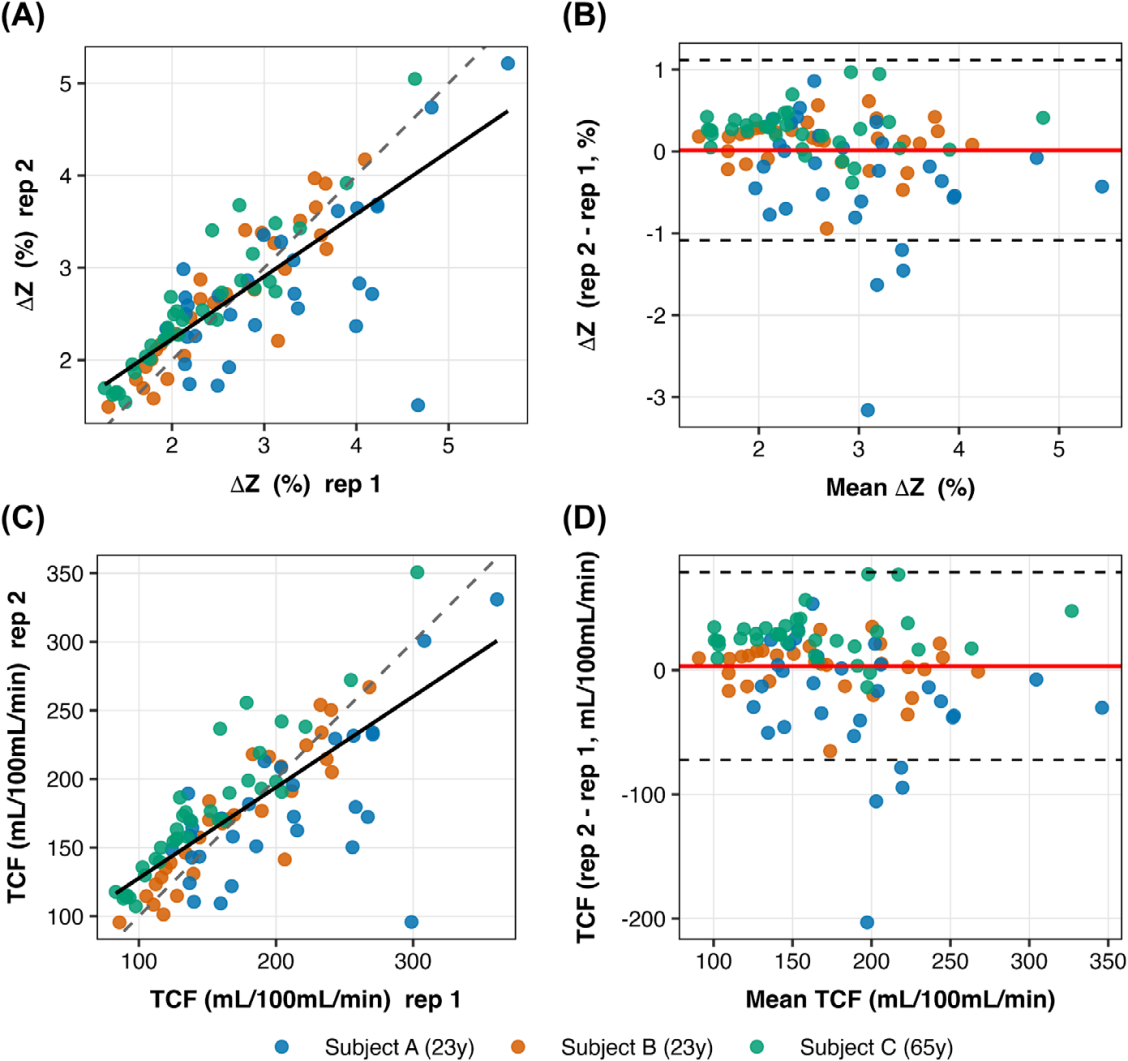
Test–retest reproducibility of MISL‐derived measurements. (A) Scatter plot of ΔZ (%) between two repeated scans across all atlas‐based ROIs from three subjects. The solid line represents the linear regression, and the dashed line represents identity. (B) Bland–Altman plot for ΔZ showing mean bias (red) and 95% limits of agreement (dashed). (C) Scatter plot of TCF (mL/100 mL/min) between two repeated scans. (D) Bland–Altman plot for TCF.

The ΔZ and TCF values were extracted from each atlas‐based ROI and compared between sessions for the three volunteers who were scanned twice. As shown in Figure 7, both parameters demonstrated good agreement across repeated scans. Specifically, ΔZ showed ICC [1, 2] = 0.784 (95% CI: 0.693–0.851) and *R* = 0.792 with a Bland–Altman bias of 0.017% (LoA = [−1.084, 1.117]). TCF showed ICC [1, 2] = 0.754 (95% CI: 0.653–0.829) and *R* = 0.761 with a Bland–Altman bias of 3.28 mL/100 mL/min (LoA = [−72.31, 78.88]). These results demonstrate good test–retest reliability for both ΔZ and TCF across brain regions, supporting the robustness of MISL‐derived tissue‐CSF exchange measurements.

### 3.6 Age‐Related Decline in Tissue‐CSF Exchange Measured by MISL

**FIGURE 8:**
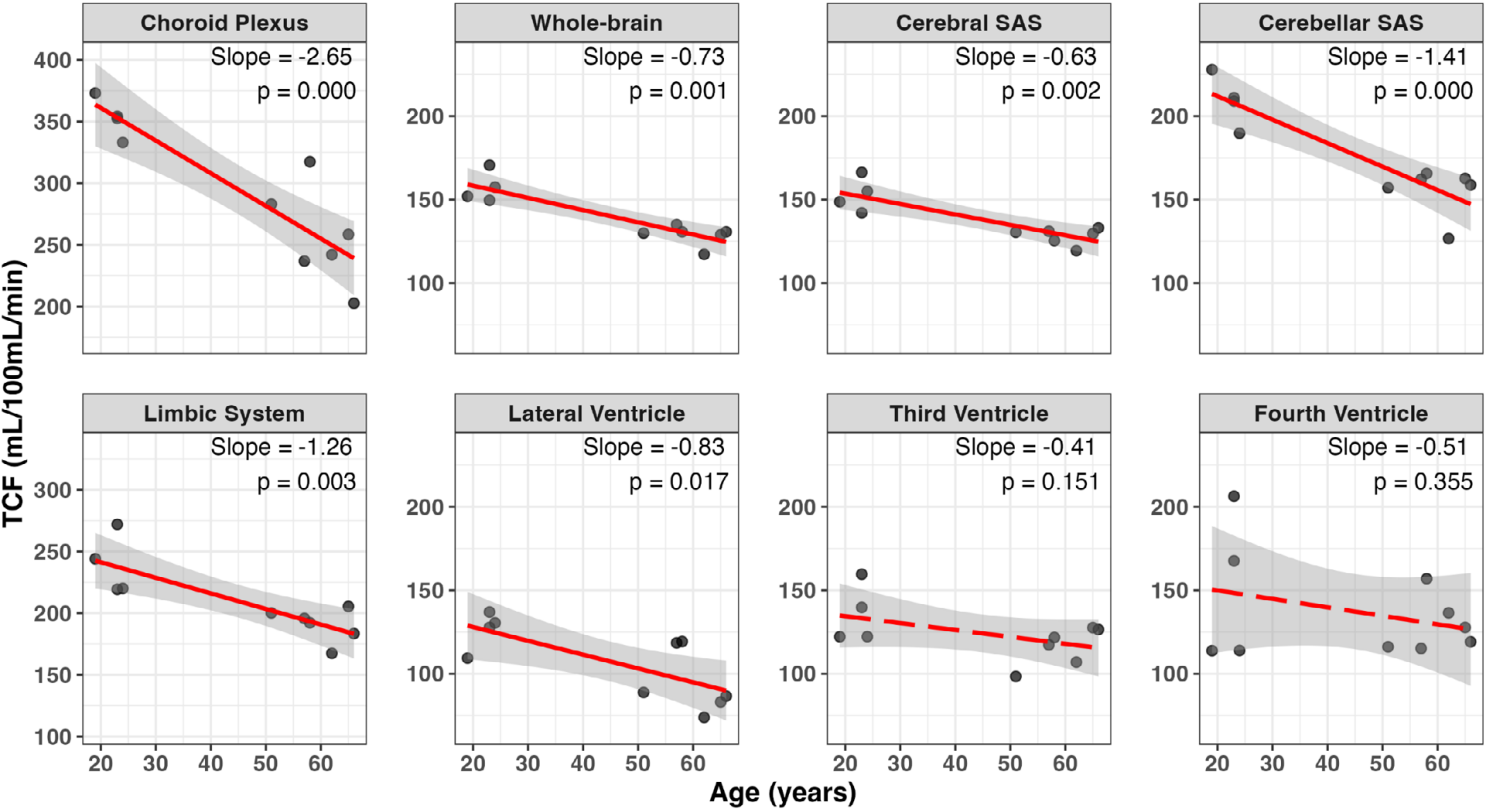
Linear regression plots show age‐dependent decline in tissue‐to‐CSF flow rate (TCF) across various CSF compartments (*n* = 10), including the choroid plexus, CSF arund the limbic system, whole‐brain CSF, cerebral subarachnoid space (SAS), cerebellar SAS, lateral ventricles, third ventricle, and fourth ventricle. The age‐dependent slope and corresponding *p*‐values for each region are indicated across seven CSF regions. All regions, except the third and fourth ventricles, show strong age dependence.

We assessed the tissue‐CSF exchange rate TCF across multiple CSF compartments and age groups. Linear regression analysis in Figure 8 revealed significant age‐associated decreases in TCF within the CP (slope = −2.91 mL/100 mL/min/year, *p* < 0.001), CSF around limbic system (slope = −1.26 mL/100 mL/min/year, *p* = 0.003), whole‐brain CSF (−0.73 mL/100 mL/min/year, *p* < 0.001), cerebral SAS (−0.63 mL/100 mL/min/year, *p* = 0.002), cerebellar SAS (−1.41 mL/100 mL/min/year, *p* < 0.001), and lateral ventricle (−0.83 mL/100 mL/min/year, *p* = 0.017). The third and fourth ventricles showed weaker, non‐significant associations with age (−0.41 and −0.51 mL/100 mL/min/year, respectively). These findings indicate that tissue‐CSF exchange dynamics decline significantly with age, particularly in the CP, cerebellar SAS, and CSF around limbic system. In contrast, intraventricular spaces like the third and fourth ventricles appear less sensitive to aging‐related changes in exchange.

### 3.7 Abnormal Water Exchange in Tumor and Tumor‐Associated Edema

**FIGURE 9:**
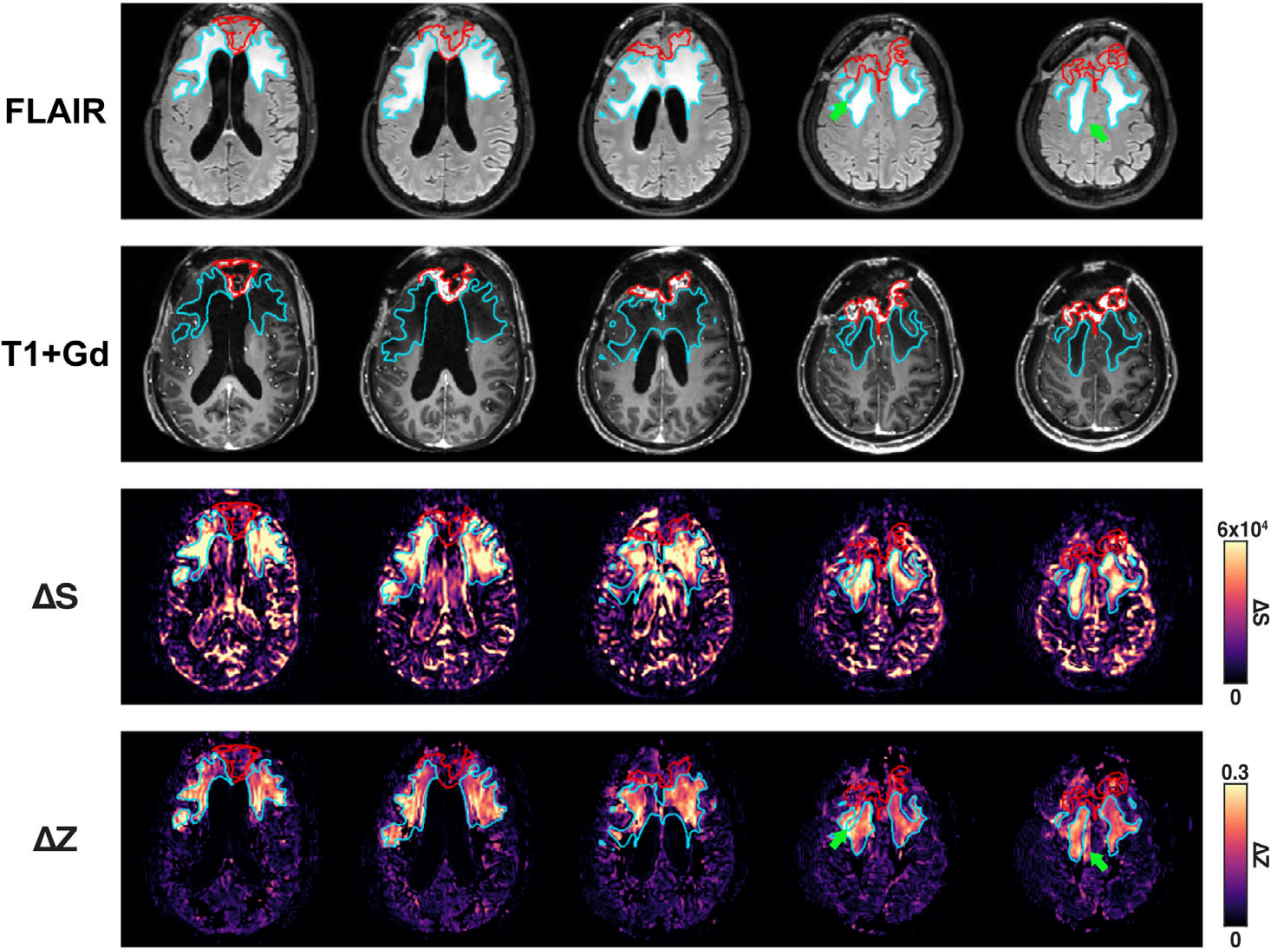
FLAIR, post‐contrast T1w, MISL ΔS, and MISL ΔZ images of a patient with brain metastases. The tumor is outlined in red and the peritumoral edema in blue. Green arrows indicate regions not hyperintense on FLAIR but showing elevated MISL ΔZ.

In a patient with brain metastases secondary to breast cancer, representative images of FLAIR, T1 post‐contrast, MISL ΔS, and MISL ΔZ are shown in Figure 9. The tumor is outlined in red and the peritumoral edema in blue. We observed stronger MISL signals in both tumor cores and peritumoral edema regions, indicating accelerated water exchange. Regions with high MISL signal largely overlapped with FLAIR hyperintensities but exhibited marked spatial heterogeneity. Interestingly, areas of elevated MISL signal were also observed beyond regions of FLAIR hyperintensity (green arrows), potentially suggesting incipient edema not yet apparent on conventional MRI.

## 4 Discussion

This study implements an off‐resonance MISL method to detect tissue‐CSF water exchange in the human brain, including the ventricles, SAS, and PVS. Simulation results and in vivo optimization demonstrated negligible contamination from DS, confirming the specificity of the method in detecting tissue‐CSF water exchange. MISL revealed widespread and rapid tissue‐CSF exchange across the brain, with particularly high exchange rates observed in the PVS and CP regions when comparing regional water exchange rates. We further found that tissue‐CSF water exchange is strongly age‐dependent, which is consistent with the animal MISL study [39] and on‐resonance MISL in the human brain [43]. As a proof‐of‐concept application, we applied MISL to assess water dynamics in metastatic brain tumors and peritumoral edema. These findings highlight the sensitivity and utility of the MISL approach for non‐invasive quantification of tissue‐CSF water exchange in the human brain at 3 T.

When applying the MISL method for quantifying tissue‐CSF water exchange, the primary confounding factor is DS. Therefore, the saturation offset, B_1_, and duration must be carefully optimized to minimize DS contamination. Off‐resonance MISL addresses this by using saturation pulses at frequency offsets well away from water, thereby greatly reducing DS. Previous studies have suggested that a short interpulse delay does not reduce labeling efficiency for slow‐exchanging protons such as those involved in MT, since these labeled protons require additional time to exchange with water [46]. Therefore, a 25 ms delay was used in the current study. This approach not only further minimizes DS effects but also reduces hardware demands, making it more suitable for clinical scanners. Simulations in pure water and in vivo validation using a subject with a left peri‐Sylvian arachnoid cyst under the current acquisition parameters demonstrate that DS is reduced to approximately 0.3%, which is about 10% of the typical MISL signal (∼2%–4%), confirming its negligible contribution. In the present study, a subject with a left peri‐Sylvian arachnoid cyst was used specifically for validation. Alternatively, DS contamination can also be estimated in patients with hydrocephalus or in elderly subjects by examining regions within enlarged ventricles. Currently, we use a long‐TE TSE readout with high CS factor. The 8‐fold CS acceleration was intended to balance SNR with motion sensitivity. A higher CS factor reduces the number of shots per volume (two shots in this study), which helps minimize motion artifacts. While inter‐volume motion across the 18 control/label pairs can be effectively corrected through rigid‐body registration during post‐processing, motion occurring within each shot for each volume is much more challenging to correct.

For the ΔZ maps, a CSF mask was applied because ΔZ is inherently sensitive to noise outside CSF regions. Specifically, ΔZ maps (Equation 3) are derived from a long‐TE TSE readout designed to suppress tissue signal; consequently, residual tissue magnetization outside the CSF, that is, $S_{\text{Control}}$, is extremely small, leading to dramatically increased noise in these regions in the ΔZ maps. As a result, ΔZ values outside the CSF mask are unreliable for interpretation and were therefore not displayed in the ΔZ maps (Figures 4 and 5). The current CSF mask was generated using a relatively simple threshold‐based approach on high‐resolution T2‐weighted images, which not fully capture the spatial extent of perivascular signal, particularly those small PVS. Consequently, signal changes in tissue adjacent to the PVS may appear in the ΔS maps but are excluded from ΔZ visualization due to masking (Figure 5). The ΔS signal in tissue ouside the CSF and PVS may still arise from other sources and therefore require careful examination.

The majority of MISL signals observed in the SAS ranged from approximately 2%–4%. These values are slightly lower than those reported for on‐resonance MISL (typically 3%–5%), as the parenchymal labeling efficiency achieved by on‐resonance MT pulses is inherently higher than that of off‐resonance MT [47, 48], resulting in higher MISL signal. A direct, side‐by‐side comparison of the two approaches in terms of SNR and to B_0_/B_1_ inhomogeneity is necessary to determine which method is more suitable for future clinical applications. Our MISL signals are more than double the signal obtained using T2‐based labeling methods (0.5%–2%) [42]. This improved sensitivity enables high‐resolution water exchange mapping at 2 mm resolution, even at the single‐subject level. These findings align with prior animal studies, where MISL signal intensity was more than three times higher than that obtained using T1 or ADC‐based labeling approaches [39, 41]. The measured TCF in humans is approximately ∼180 mL/100 mL/min, whereas in mice it is around 800 mL/100 mL/min—roughly 4.4 times higher [39]. Interestingly, this ratio is comparable to that of cerebral blood flow (∼4), which is ∼50 mL/100 mL/min in humans [49] versus ∼200 mL/100 mL/min in mice [50, 51]. Note that MISL ΔS and ΔZ maps are highly dependent on scanner hardware and labeling efficiency, making cross‐study comparisons challenging. In contrast, the TCF metric is theoretically independent of these factors, providing a more robust basis for comparison.

Significant regional variations in TCF were observed across the brain (Figure 6), aligning with patterns previously reported using T2‐based labeling methods [42]. Notably, rapid water exchange was also detected in the PVS, where TCF values were substantially higher (∼8.4%) compared to those in the ventricles and SAS (2%–4%), highlighting the PVS as a highly dynamic interface for tissue‐CSF water transport. Although the small size of the PVS may introduce partial‐volume effects from surrounding parenchyma, the long‐TE readout effectively suppresses tissue signal, as demonstrated in Supporting Figure S1. As shown in Figure 5, long‐TE T2w images can only identify the PVS structures, but MISL is able to evaluate the tissue‐CSF water exchange within PVS. Given that the PVS plays a critical role in the glymphatic system by mediating fluid transport between the CSF and ISF, the ability of MISL to non‐invasively evaluate water exchange dynamics within the PVS provides functional information that conventional T2w images cannot capture and offers a promising tool for assessing glymphatic function. Aging is known to reduce glymphatic influx (CSF‐to‐tissue flow), largely due to glial‐vascular changes, such as loss of perivascular AQP4 localization and decline in vascular pulsatility [52]. These same factors can also impair the efflux (tissue‐to‐CSF exchange) that MISL detects, leading to a global decline in interstitial fluid movement and waste clearance. Therefore, the observed age‐related decrease in TCF likely reflects a reduction in overall glymphatic transport efficiency, consistent with prior reports of impaired glymphatic function in the aging brain.

It is important to note that TCF quantification can be influenced by several factors, including CSF T_1_, tissue $R_{1\rho }$, and MT effects (Equation 4). In the current study, we used constant values for CSF T_1_ and R_1_ρ, and assumed an average MT value across the brain. Although these parameters may vary slightly with age and across different brain regions, previous studies have shown that such variations are relatively small. Therefore, using global representative values provides a reasonable approximation for our model. In a spin‐locker study, the mean $T_{1\rho }$ was 78.4 ms for cortical GM with an age slope of −0.051 ms/year, 75.6 ms for juxtacortical WM with a slope of 0.002 ms/year, and 77.9 ms for Juxtacortical WM tracts with a slope of 0.048 ms/year [53]. The T_1_ of CSF has been reported to be approximately 4454 ms in the ventricles and 4309 ms in the subarachnoid space [54]. We therefore assumed a representative value of T_1,CSF_ = 4350 ms in our model. To our knowledge, no prior studies have systematically investigated how CSF T_1_ varies with age. MT effects are smaller in GM than in WM. However, the difference is modest; typical magnetization transfer ratios are approximately 30% in GM and 33% in WM [55].

The potential sources of the MISL signal are illustrated in Figure 1A. Due to the high water exchange rates measured, it was initially believed that the primary contributor to the MISL signal was CSF exchange with the ependymal layer, particularly in regions densely lined with epithelial cells such as the CP [39]. This structure, with its extensive surface area and active transport function, consistently shows the strongest MISL signals. However, animal studies using AQP4 inhibitors have revealed that ISF to CSF exchange also contributes significantly to the MISL signal [40]. This finding is further supported by results from selectively labeling ISF [43]. The high MISL signal in PVS also reflects active fluid exchange from ISF into the surrounding PVS. It is important to note that traditional ASL methods with long‐TE readouts primarily detect water transport from blood to ISF, and eventually into the ventricles or SAS [37, 38]. This multi‐step process is time‐consuming and results in significant decay of the labeled blood signal. Moreover, ASL captures only a small portion of the total tissue‐to‐CSF exchange, i.e., blood to CSF, leading to very low signal levels, typically around 0.1%–0.3% [37, 38]. In contrast, MISL directly labels ISF in brain parenchyma and ependymal layer that exchanges with CSF, resulting in a significantly stronger signal.

Water circulation dysfunction in patients with brain tumors can be assessed using the MISL MRI. In this study, we observed stronger MISL signals in both tumor cores and peritumoral edema regions, indicating accelerated water exchange. Several histopathological studies have reported upregulation of aquaporin‐4 (AQP4) in these regions, particularly in peritumoral edema (AQP4 expression: ∼4.4 in tumor vs. ∼27.3 in edema), which facilitates transmembrane and interstitial water movement [52, 56]. This upregulation likely contributes to the stronger MISL signal observed in these regions. Compared with the conventional FLAIR image, which primarily reflects total water content, the MISL signal may provide complementary information on water exchange and early edema formation. Most regions with high MISL signal overlapped with FLAIR hyperintensities. This is expected in vasogenic edema, where BBB disruption and AQP4 upregulation increase extracellular volume and prolonged T_2_. It allows extracellular water to remain detectable with our long‐TE readout, enabling MISL to capture the intracellular‐extracellular water exchange process. Within FLAIR‐hyperintense areas, MISL showed spatial heterogeneity, plausibly reflecting local differences in water dynamics, vascular permeability, or AQP4 distribution, which may serve as biomarkers of edema progression or treatment response. Interestingly, areas with elevated MISL signal were also detected beyond FLAIR hyperintensities, suggesting that MISL may identify incipient edema before it becomes visible on conventional MRI. This highlights the potential of MISL to capture the dynamic process of edema development, rather than just its end‐state accumulation.

This study has several limitations. First, MISL is sensitive to subject motion, particularly at tissue‐CSF interfaces, where residual motion can blur boundaries and bias exchange estimates. Future implementations with background suppression, navigator‐based motion correction, and prospective motion tracking may mitigate this issue. Second, the compartmental origin of the MISL signal is not fully resolved. The relative contributions from ISF, the ependymal layer, and pia mater remain unknown. Future work using parameter sweeps (labeling and post‐labeling duration) and histologic correlation will be needed to quantify each component's contribution. Third, our tumor data are cross‐sectional, which limits inference about temporal evolution. Longitudinal MISL will be necessary to determine whether early exchange abnormalities predict edema progression or treatment response.

## 5 Conclusions

This study demonstrates that MISL is a sensitive and specific technique for mapping tissue‐CSF water exchange in the human brain. By selectively labeling parenchymal water without direct saturation of CSF, MISL captures dynamic water transport processes across ventricles, subarachnoid space, and perivascular space. MISL's ability to evaluate tissue‐CSF water exchange within PVS and the observed age‐related reductions in exchange underscore its potential utility in both physiological and pathological contexts. Compared to conventional MRI, MISL may capture the dynamic process of edema development. Future studies will aim to establish normative values, validate predictive capability in longitudinal cohorts, and expand clinical translation across disease models.

## Funding

This work was supported by National Institutes of Health, P30AG066507, P41EB031771, R01AG080104, R01HL149742.

## Supporting information


**Data S1:** Supporting Information.

## Data Availability

The data that support the findings of this study are available on request from the corresponding author. The data are not publicly available due to privacy or ethical restrictions.
