# Supplementary material for: Rapid Tissue‐CSF Water Exchange in the Human Brain Revealed by Magnetization Transfer Indirect Spin Labeling
Source: Magn Reson Med. 2026 Feb 9;95(6):3270–83. doi: 10.1002/mrm.70298 (PMC13049270; doi:10.1002/mrm.70298)
Supplement: Supplementary file 1 — Data S1: Supporting Information. [file MRM-95-3270-s001.pdf]

Supplementary Material

MISL Quantification Theory

A standard single-compartment kinetic model was applied to quantity the tissue-CSF exchange, assuming instantaneous exchange of labeled spins from tissue to CSF. The assumption of instantaneous exchange of labeled spins from tissue to CSF is based on our previous mouse brain study (1), in which a pulsed labeling approach demonstrated rapid CSF–tissue water exchange with transit times on the order of 13–56 ms. However, this pulsed labeling strategy does not translate well to human studies because exchange rates in the human brain are substantially slower. Consequently, the transit time cannot yet be directly measured in humans using this approach. Nevertheless, we expect the transit time in humans to remain short, as the labeled tissue is in close proximity to the CSF. A more careful examination and validation of this assumption in the human brain will be an important focus of future studies. The observed CSF signal reduction is described by the difference between the sum over the series of delivered magnetization units to CSF from tissue water (the tissue input function, TIF), and the clearance of the magnetization by CSF flow and relaxation of the CSF (the impulse residue function, IRF) (2-4). When a magnetization transfer (MT) pulse train is applied, the dependence of tissue water labeling (ΔZ^{Tissue}) on the saturation time (tsat) can be described by a mono-exponential recovery function based on the principles of rotating frame theory: (5,6)

$$TIF = \Delta Z^{Tissue}(t) = \alpha \cdot \frac{\text{TCF}}{6000} \cdot (1 - e^{-R_{1\rho,tissue} \cdot t_{sat}}) \qquad (S1),$$

Where α is the labeling efficiency of the MT pulse on the tissue water, R1ρ, tissue is the water relaxation rate of parenchyma in the rotating frame. The tissue-to-CSF flow (TCF) rate is expressed in units of tissue water volume delivered per 100 unit volume of CSF per minute, mL/100 mL/min. The observed CSF signal reduction (ΔZ) is the convolution of the TIF and the IRF functions, i.e.,

$$\Delta Z = \Delta Z^{Tissue}(t) \otimes e^{-\frac{t_{sat}}{T_{1eff}}} \qquad (S2),$$

Note that T1eff is an apparent relaxation time for a monoexponential process, which accounts for the "true" T1 relaxation of the CSF and clearance of labeled CSF by the CSF outflow. In our study, T_{1eff} was approximated as the T1 relaxation time of the CSF, i.e. $T_{1eff} \approx T_{1csf}$. Then,

$$\Delta Z = \int_0^{t_{sat}} \alpha \cdot \frac{\text{TCF}}{6000} \cdot \left(1 - e^{-R_{1\rho,tissue} \cdot t'}\right) e^{-\frac{(t_{sat} - t')}{T_{1\text{csf}}}} dt' \qquad (S3),$$

$$=\int_0^{t_{sat}} \alpha \cdot \frac{\mathrm{TCF}}{6000} \cdot e^{-\mathrm{R}_{1\mathrm{CSF}}(t_{sat}-t')} dt' - \int_0^{t_{sat}} \alpha \cdot \frac{\mathrm{TCF}}{6000} \cdot e^{-R_{1\rho,tissue} \cdot t'} e^{-\mathrm{R}_{1\mathrm{CSF}}(t_{sat}-t')} dt' \qquad (S4),$$

$$= \alpha \cdot \frac{\mathrm{TCF}}{6000} \cdot e^{-\mathrm{R}_{1\mathrm{CSF}} t_{sat}} \left(\int_0^{t_{sat}} e^{\mathrm{R}_{1\mathrm{CSF}} t'} dt' - \int_0^{t_{sat}} e^{-R_{1\rho,tissue} \cdot t'} e^{\mathrm{R}_{1\mathrm{CSF}} \cdot t'} dt' \right) \qquad (S5),$$

$$= \alpha \cdot \frac{\mathrm{TCF}}{6000} \cdot e^{-\mathrm{R}_{1\mathrm{CSF}} t_{sat}} \left(\frac{e^{\mathrm{R}_{1\mathrm{CSF}} \cdot t_{sat}}}{\mathrm{R}_{1,\mathrm{CSF}}} - \frac{1}{\mathrm{R}_{1,\mathrm{CSF}}} + \frac{1}{\mathrm{R}_{1,\mathrm{CSF}} - R_{1\rho,tissue}} - \frac{e^{(\mathrm{R}_{1,\mathrm{CSF}} - R_{1\rho,tissue}) \cdot t_{sat}}}{\mathrm{R}_{1,\mathrm{CSF}} - R_{1\rho,tissue}} \right) \qquad (S6),$$

$$= \alpha \cdot \frac{\mathrm{TCF}}{6000} \cdot \frac{1}{\mathrm{R}_{1,\mathrm{CSF}}} \left(1 - e^{-\mathrm{R}_{1\mathrm{CSF}} \cdot t_{sat}} + \frac{\mathrm{R}_{1,\mathrm{CSF}} \cdot e^{-\mathrm{R}_{1,\mathrm{CSF}} \cdot t_{sat}}}{\mathrm{R}_{1,\mathrm{CSF}} - R_{1\rho,tissue}} - \frac{\mathrm{R}_{1,\mathrm{CSF}} \cdot e^{-R_{1\rho,tissue} \cdot t_{sat}}}{\mathrm{R}_{1,\mathrm{CSF}} - R_{1\rho,tissue}} \right) \qquad (S7),$$

$$= \alpha \cdot \frac{\mathrm{TCF}}{6000} \cdot \frac{1}{\mathrm{R}_{1,\mathrm{CSF}}} \left(1 + \frac{R_{1\rho,tissue} \cdot e^{-\mathrm{R}_{1,\mathrm{CSF}} \cdot t_{sat}}}{\mathrm{R}_{1,\mathrm{CSF}} - R_{1\rho,tissue}} - \frac{\mathrm{R}_{1,\mathrm{CSF}} \cdot e^{-R_{1\rho,tissue} \cdot t_{sat}}}{\mathrm{R}_{1,\mathrm{CSF}} - R_{1\rho,tissue}} \right) \qquad (S8),$$

Let $\mathrm{R}_{1,\mathrm{app}} = R_{1\rho,tissue} - \mathrm{R}_{1,\mathrm{CSF}}$, then

$$\Delta \mathrm{Z} = \alpha \cdot \frac{\mathrm{TCF}}{6000} \cdot \frac{1}{\mathrm{R}_{1,\mathrm{CSF}}} \left(1 + \mathrm{e}^{-\mathrm{R}_{1\rho,\mathrm{tissue}} \cdot t_{sat}} \cdot \frac{\mathrm{R}_{1,\mathrm{CSF}}}{\mathrm{R}_{1,\mathrm{app}}} - \mathrm{e}^{-\mathrm{R}_{1,\mathrm{CSF}} \cdot t_{sat}} \cdot \frac{\mathrm{R}_{1\rho,\mathrm{tissue}}}{\mathrm{R}_{1,\mathrm{app}}} \right) \qquad (S9),$$

It needs to point out that the current theoretical framework is based on a unidirectional exchange assumption. Although water exchange between tissue and CSF is inherently bidirectional, the MISL experiment selectively labels tissue water, while CSF water remains unlabeled. Consequently, only tissue-to-CSF transfer contributes to the observed CSF signal change, making the transport of labeled magnetization effectively unidirectional. The resulting CSF signal reduction is on the order of only 2–3% of the total water signal; therefore, backward exchange from CSF to tissue carries negligible labeled magnetization and does not measurably affect ΔZ. This assumption is further supported by our previous mouse brain study (1), in which CSF was selectively labeled using a long-T_2 preparation module and negligible labeled spins were observed in brain tissue.

Assessment of Tissue Signal Contribution to PVS ΔZ

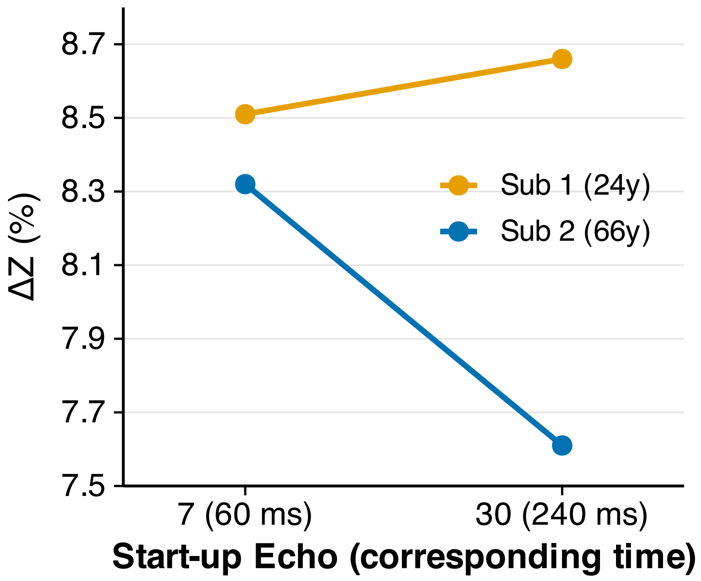

Figure S1. Comparison of MISL ΔZ at start-up echoes of 7 (60 ms) and 30 (240 ms) in two subjects.

To evaluate whether the MISL ΔZ observed in the perivascular space (PVS) could arise from incomplete tissue signal suppression in the TSE readout, we compared MISL ΔZ signals acquired with start-up times of 60 ms and 240 ms in two subjects. The ΔZ in the PVS showed minimal change between the two start-up times (8.5% at 60 ms vs. 8.6% at 240 ms in one subject; 8.3% vs. 7.6% in the other). The absence of consistent ΔZ reduction at shorter start-up times indicates that the PVS signal is not primarily driven by unsuppressed tissue signal early in the echo train. Instead, the data suggest that the observed ΔZ reflects tissue-CSF water exchange signal, with neglectable contributions from residual tissue signal.

References:

1. Li AM, Xu J. Cerebrospinal fluid-tissue exchange revealed by phase alternate labeling with null recovery MRI. Magn Reson Med 2022;87(3):1207-1217.
2. Calamante F. Arterial input function in perfusion MRI: a comprehensive review. Progress in Nuclear Magnetic Resonance Spectroscopy 2013:DO: 10.1016/j.pnmrs.2013.1004.1002.

3. Wong EC, Buxton RB, Frank LR. A theoretical and experimental comparison of continuous and pulsed arterial spin labeling techniques for quantitative perfusion imaging. Magn Reson Med 1998;40(3):348-355.
4. Qin Q, Huang AJ, Hua J, Desmond JE, Stevens RD, van Zijl PCM. Three-dimensional whole-brain perfusion quantification using pseudo-continuous arterial spin labeling MRI at multiple post-labeling delays: accounting for both arterial transit time and impulse response function. NMR Biomed 2014;27(2):116-128.
5. Jin T, Autio J, Obata T, Kim S-G. Spin-locking versus chemical exchange saturation transfer MRI for investigating chemical exchange process between water and labile metabolite protons. Magn Reson Med 2011;65(5):1448-1460.
6. Zaiss M, Bachert P. Chemical exchange saturation transfer (CEST) and MR Z-spectroscopy in vivo: a review of theoretical approaches and methods. Phys Med Biol 2013;58(22):R221-269.
